# No neutralizing effect of pre-existing tick-borne encephalitis virus antibodies against severe acute respiratory syndrome coronavirus-2: a prospective healthcare worker study

**DOI:** 10.1038/s41598-021-03685-y

**Published:** 2021-12-17

**Authors:** Philipp Kohler, Hulda R. Jonsdottir, Lorenz Risch, Pietro Vernazza, Rahel Ackermann-Gäumann, Christian R. Kahlert

**Affiliations:** 1 Division of Infectious Diseases and Hospital Epidemiology, Cantonal Hospital St Gallen, St Gallen, Switzerland; 2 Spiez Laboratory, Federal Office for Civil Protection, Spiez, Switzerland; 3 Swiss National Reference Centre for Tick-Transmitted Diseases, Spiez, Switzerland; 4 Department of Rheumatology, Immunology, and Allergology, Inselspital University Hospital, Bern, Switzerland; 5 Department of BioMedical Research, University of Bern, Bern, Switzerland; 6 Labormedizinisches Zentrum Dr. Risch, Buchs, Switzerland; 7 Department of Infectious Diseases and Hospital Epidemiology, Children’s Hospital of Eastern Switzerland, St.Gallen, Switzerland

**Keywords:** Viral infection, Antibodies

## Abstract

Certain immunizations including vaccination against tick-borne encephalitis virus (TBEV) have been suggested to confer cross-protection against severe acute respiratory syndrome coronavirus-2 (SARS-CoV-2). Within a prospective healthcare worker (HCW) cohort, we assessed the potentially protective role of anti-TBEV antibodies against SARS-CoV-2 infection. Among 3352 HCW, those with ≥ 1 previous TBEV vaccination (n = 2018, 60%) showed a reduced risk of SARS-CoV-2 seroconversion (adjusted odds ratio: 0.8, 95% CI: 0.7–1.0, P = 0.02). However, laboratory testing of a subgroup of 26 baseline and follow-up samples did not demonstrate any neutralizing effect of anti-TBEV antibodies against SARS-CoV-2 in live-virus neutralization assay. However, we observed significantly higher anti-TBEV antibody titers in follow-up samples of participants with previous TBEV vaccination compared to baseline, both TBEV neutralizing (p = 0.001) and total IgG (P < 0.0001), irrespective of SARS-CoV-2 serostatus. Based on these data, we conclude that the observed association of previous TBEV vaccination and reduced risk of SARS-CoV-2 infection is likely due to residual confounding factors. The increase in TBEV follow-up antibody titers can be explained by natural TBEV exposure or potential non-specific immune activation upon exposure to various pathogens, including SARS-CoV-2. We believe that these findings, although negative, contribute to the current knowledge on potential cross-immunity against SARS-CoV-2 from previous immunizations.

## Introduction

The burden of the coronavirus disease 2019 (COVID-19) pandemic shows substantial geographic variation^1^. A range of potential causes for this have been proposed, including differences in the implementation of non-pharmaceutical preventive measures (e.g. movement restrictions, face masks, school closures), demographic (e.g. population age and density), social (e.g. household structures), or environmental (temperature, humidity, air pollution) factors^2^.

Another intriguing hypothesis is that pre-existing immunity against other pathogens might confer a partial resistance to severe acute respiratory syndrome coronavirus 2 (SARS-CoV-2). Several candidates have been proposed, including previous exposure to endemic coronaviruses^3^, malaria^4^, seasonal influenza^5^, Bacillus Camille-Guerin vaccination^6^, measles vaccination^7^, and flaviviruses^8^. Data from Brazil show that geographic regions with high dengue virus (DENV) burden have reported fewer COVID-19 cases^9^ and COVID-19 patients previously infected with DENV had a lower case fatality rate compared to those without previous infection^8^. Furthermore, structural similarities between SARS-CoV-2 and DENV leading to potential antigenic cross-reactivity have been postulated^10^. Other members of the flaviviridae family include Japanese encephalitis virus (JEV) and tick-borne encephalitis virus (TBEV) and it has been hypothesized that differential burden of COVID-19 could—at least partly—be attributable to different national vaccination strategies for JEV (in Asian countries) and TBEV (in European countries)^11^.

Combined, these findings prompted us to investigate any potential cross-immunity against SARS-CoV-2 mediated by anti-TBEV antibodies. First, we assessed the association between self-reported TBEV vaccination and SARS-CoV-2 seropositivity within a large prospective healthcare worker (HCW) study. Second, we analysed baseline and follow-up samples from HCW to evaluate whether anti-TBEV antibodies showed any cross-neutralizing effect against SARS-CoV-2 and whether SARS-CoV-2 infection could increase titers of pre-existing anti-TBEV antibodies.

## Results

### Previous TBEV vaccination and SARS-CoV-2 seroconversion

The HCW cohort included 3352 individuals, whereof 633 (19%) had positive SARS-CoV-2 serology. Among these 633, 352 (56%) reported a previous TBEV vaccination compared to 1666 (61%) out of 2719 with a negative SARS-CoV-2 serology. Previous TBEV vaccination was therefore associated with decreased risk of SARS-CoV-2 seroconversion with an odds ratio (OR) of 0.8 and a 95% confidence interval (CI) of 0.7–0.9 (P = 0.009). After adjusting for predefined variables, the association remained unchanged with an adjusted OR (aOR) of 0.8; 95% CI 0.7–1.0 (P = 0.02, Table 1).

**Table 1:** Characteristics of SARS-CoV-2 seropositive and seronegative individuals from a prospective healthcare worker cohort and results of logistic regression analyses.

Total n = 3352	Seropositive	Seronegative	Univariable			Multivariable		
	n = 633	n = 2719	OR	95% CI	P-Value	aOR	95% CI	P-Value
TBEV vaccination	352 (56%)	1666 (61%)	0.79	0.67–0.94	0.009	0.81	0.68–0.97	0.021
Age (mean, SD)	39 (11.8)	41 (11.5)	0.99	0.98–0.99	< 0.001	0.99	0.98–0.99	0.002
Female (n = 3331)	500 (79%)	2116 (78%)	1.06	0.86–1.32	0.573	0.86	0.68–1.08	0.196
**Profession**								
Physician	89 (14%)	470 (17%)	Ref			Ref		
Nurse	359 (57%)	1086 (40%)	1.75	1.35–2.26	< 0.001	1.86	1.49–2.33	< 0.001
Other^a^	185 (29%)	1163 (43%)	0.84	0.64–1.11	0.213	1.08	0.79–1.46	0.64
Patient contact (n = 3149)	526 (83%)	2020 (74%)	1.7	1.36–2.13	< 0.001	1.25	0.96–1.62	0.100

^a^includes professions without patient contact.

*aOR* adjusted Odds Ratio, *CI* Confidence Interval, *Ref* Reference category for logistic regression, *SARS-CoV-2* Severe Acute Respiratory Syndrome Coronavirus 2, *SD* Standard Deviation, *TBEV* Tick-Borne Encephalitis Virus.

### No cross-neutralization of TBEV antibodies against SARS-CoV-2

We analysed 26 baseline (T1, March/April 2020) and follow-up (T2, August/September 2020) samples divided into three groups defined above. Group characteristics are summarized in Table 2.

**Table 2:** Characteristics of 26 healthcare workers and their TBEV/SARS-CoV-2 serostatus at time points T1 and T2.

	Size	Female	Median age (years)	TBEV vaccination	TBEV antibodies (T1;T2)	SARS-CoV-2 antibodies (T1;T2)
Group 1	4	3 (75%)	31.3	Yes	4 (100%); 4 (100%)	0 (0%); 4 (100%)
Group 2	17	10 (59%)	40.8	Yes	16 (94%); 17 (100%)	0 (0%); 0 (0%)
Group 3	5	3 (60%)	43.1	No	1 (20%); 1 (20%)	0 (0%); 0 (0%)

*SARS-CoV-2* Severe Acute Respiratory Syndrome Coronavirus-2, *TBEV* Tick-Borne Encephalitis Virus.

**Figure 1:**
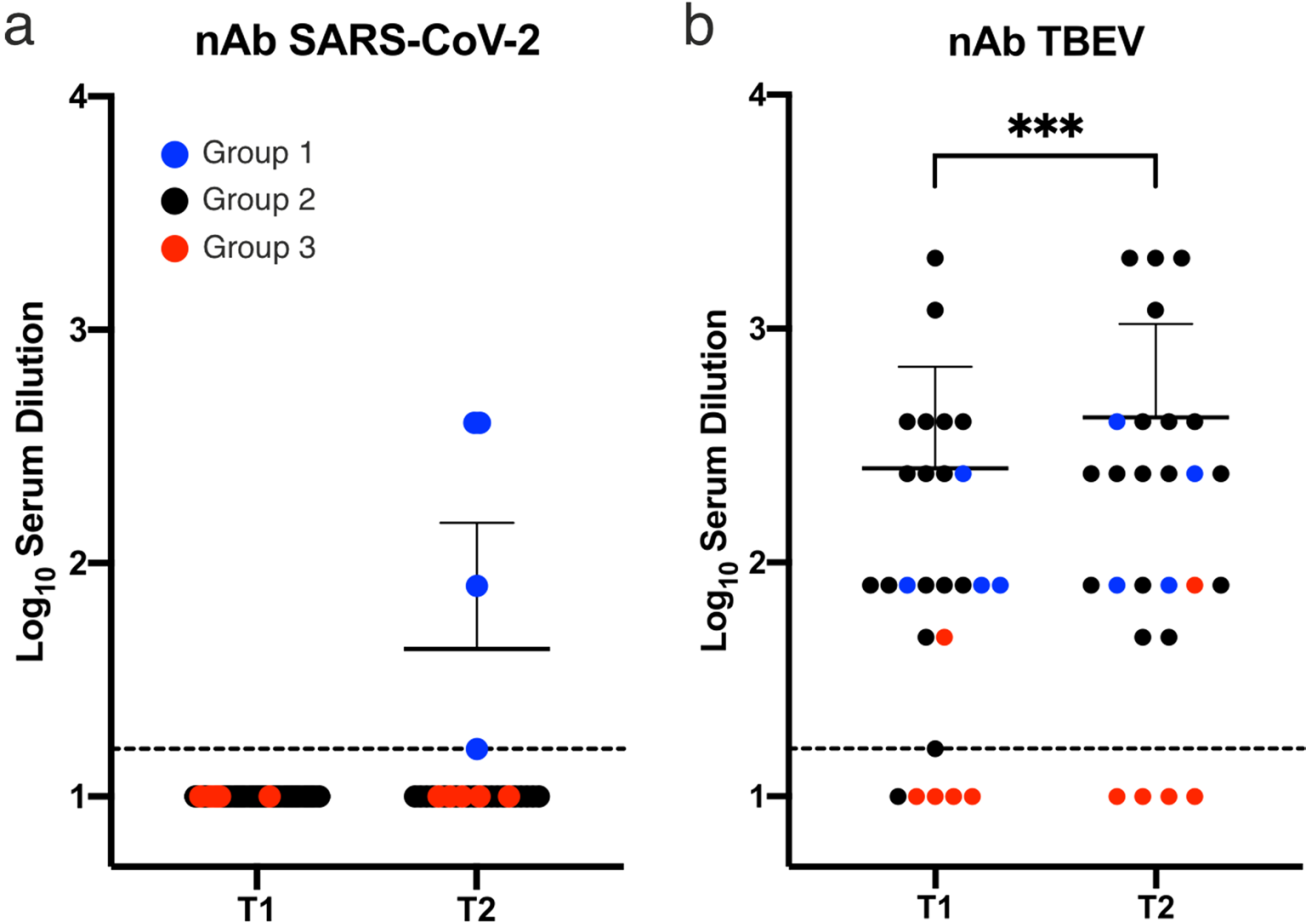
Serum neutralization test (SNT) revealed no cross-protection between SARS-CoV-2 and TBEV. (**a**) Neutralization against SARS-CoV-2 was only observed in convalescent patients (4/4, blue) and no cross-reactivity was observed in individuals vaccinated against TBEV (black). (**b**) Neutralization of TBEV was observed in almost all individuals vaccinated against TBEV at T1 (16/17, black) and increased significantly between sampling points (T2, 17/17); ***p = 0.001. Dashed line: Limit of detection, 1:16 serum dilution. Non-neutralizing samples are assigned the value 10. *T1* March/April 2020, *T2* August/September 2020, *Group 1* blue: SARS-CoV-2 convalescents, *group 2* black: TBEV vaccinees, *group 3* red: negative control group.

As expected, all four individuals in group 1 had neutralizing antibodies against SARS-CoV-2 in their follow-up (T2), but not in their baseline (T1) sample (Fig. 1a, blue). However, none of the sera from group 2 exhibited any cross-neutralization against SARS-CoV-2 (Fig. 1a, black). Thus, anti-TBEV-antibodies did not have any direct neutralizing effect on SARS-CoV-2 in these samples. Additionally, all four individuals (100%) from group 1 and 16/17 individuals (94%) from group 2 had neutralizing antibodies against TBEV in both baseline and follow-up samples, except for one individual from group 2 who tested negative at T1 but positive at T2. Within the control (group 3), four individuals tested negative for neutralizing antibodies against TBEV as expected, whereas one tested positive at both T1 and T2 (Fig. 1b, red). As individuals were assigned to groups based on their vaccination history, this person is likely to have acquired anti-TBEV antibodies naturally. Overall, neutralizing TBEV antibody titers were significantly higher in the follow-up samples (T2) than in the baseline samples (T1) (P < 0.0001). However, this trend was observed in both groups 1 and 2, therefore these results do not allow for any conclusions about a triggered TBEV antibody response due to a recent SARS-CoV-2 infection. Furthermore, an increase in TBEV neutralizing antibody titer was only observed in 50% of positive samples (11/22).

### Significant increase in anti-TBEV IgG titer in follow up samples

**Figure 2:**
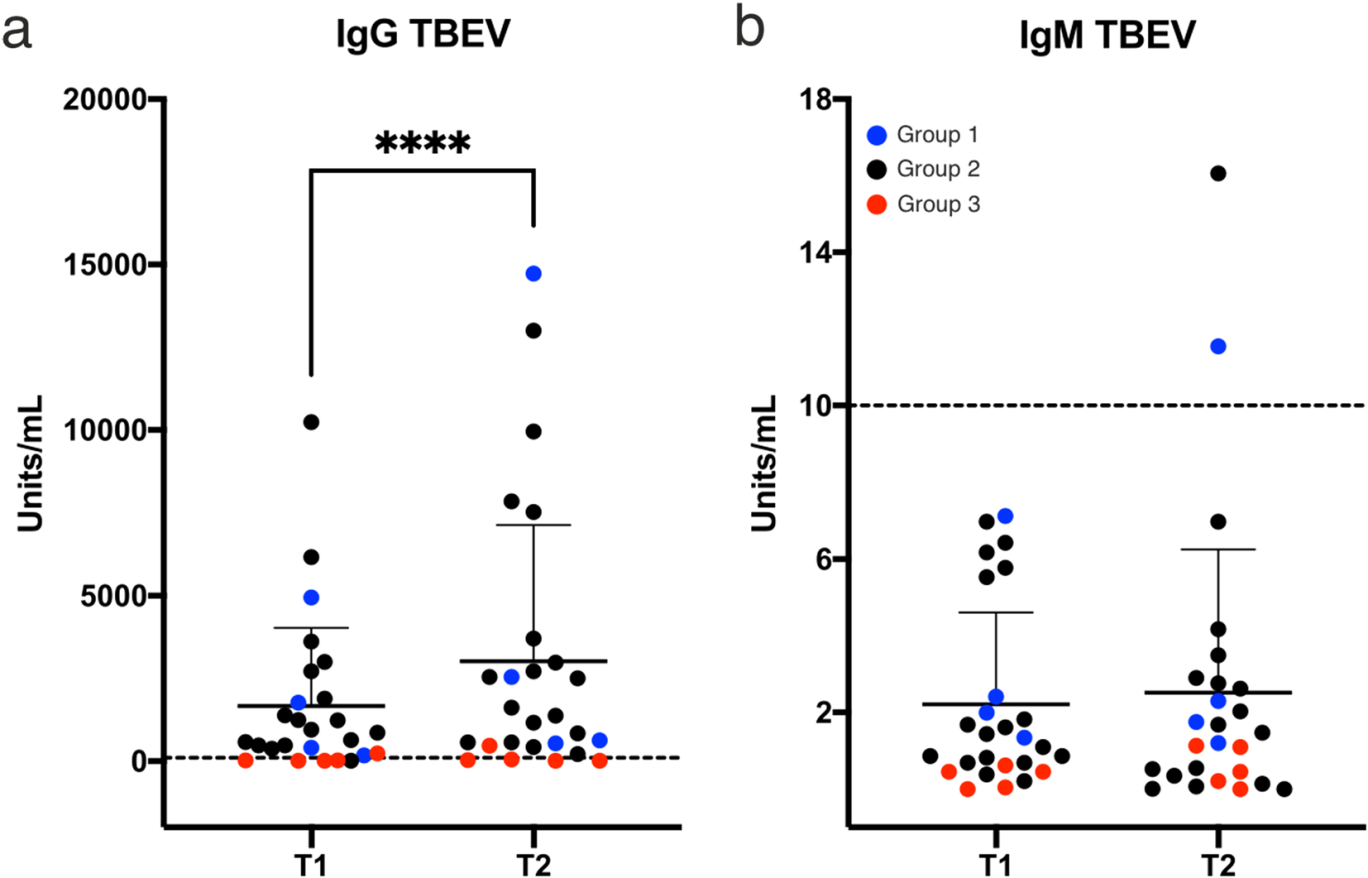
Significant increase was observed in anti-TBEV IgG antibodies in follow up samples*.* (**a**) Anti-TBEV IgG antibodies were significantly increased in follow up samples compared to baseline (T2, 16/22); **** p < 0.0001. (**b**) IgM seroconversion was observed in one sample with an equivocal increase in another. Dashed lines: Limit of negativity; IgG: 100U/ml, IgM: 10U/ml. *T1* March/April 2020, *T2* August/September 2020. Group 1, blue: SARS-CoV-2 convalescents, group 2, black: TBEV vaccinees, group 3, red: negative control group.

The qualitative results of anti-TBEV-IgG ELISA are in agreement with the observed serum neutralization test (SNT) results. As observed for neutralizing TBEV antibody titers, anti-TBEV IgG titers determined by ELISA were significantly higher in the follow-up samples (T2, higher titer in 16 of 22 samples) than in the baseline samples (T1), (P < 0.0001), again irrespective of SARS-CoV-2 serostatus (Fig. 2a). For IgM, we observed a trend towards decreasing antibody titers from T1 to T2 for all but two samples. For these two samples (one each within groups 1 and 2), IgM seroconversion was observed, however at low titers (Fig. 2b).

## Discussion

In the current study, we found an association between previous TBEV vaccination and decreased rates of SARS-CoV-2 seroconversion within a prospective cohort of Swiss HCWs. The observed association could not be explained by any cross-neutralizing effect of anti-TBEV antibodies on SARS-CoV-2. Unexpectedly, anti-TBEV antibody titers increased overall between baseline and follow-up samples, irrespective of SARS-CoV-2 seroconversion, albeit not in all samples. The hypothesis that pre-existing TBEV antibodies might confer cross-immunity against SARS-CoV-2 is intriguing, particularly given the availability of an effective TBEV vaccine. However, we could not explain this observation by characterizing the humoral adaptive immune response against TBEV and SARS-CoV-2, as there was no cross-neutralizing effect of anti-TBEV antibodies against SARS-CoV-2 or vice versa. Our findings are in agreement with former studies demonstrating that antibodies cross-neutralizing SARS-CoV-2 are rare^12–16^ and that cross-reactive immune memory appears limited largely to CD4^+^ T cells^17^. As our cohort is limited to serum samples, aspects of cellular immunity could not be studied in this population. We therefore assume that the observed effect might be due to confounding factors. For instance, since vaccination against TBEV is optional in Switzerland, HCW who choose this vaccination might be more health conscious than unvaccinated HCW. Furthermore, vaccinated HCW are possibly more likely to spend their spare time outdoors, which is associated with a lower risk of SARS-CoV-2 acquisition in comparison to indoor exposures^18–20^. However, a growing body of literature supports the premise of innate immune memory. The concept of trained immunity describes the long-term functional reprogramming of innate immune cells leading to an altered response towards a second challenge after return to a non-activated state^21^. It has been postulated that trained immunity induced by the bacillus Calmette Guerin (BCG, tuberculosis vaccine) adjuvants could confer protection against SARS-CoV-2^6, 22, 23^. Also, previous vaccination against pneumococci is associated with decreased risk of SARS-CoV-2 acquisition^24^. Since, as observed here for TBEV, anti-pneumococcal antibodies do not have a direct neutralising effect on SARS-CoV-2, non-specific activation of the immune system has been suspected, which is in accordance with the concept of trained immunity. Further supporting this hypothesis, other recent non-COVID-19 vaccinations (including polio, varicella, measles-mumps-rubella, seasonal influenza and others) have also been associated with a decreased risk for SARS-CoV-2 infection^25^. However, for immunity against the fusion protein of the measles virus, some evidence of cross-reaction with the receptor binding domain of SARS-CoV-2 has been proposed^25^.

Interestingly, both neutralizing capacity and IgG antibody titers against TBEV increased between baseline and follow-up samples for most HCW, irrespective of SARS-CoV-2 seroconversion. A possible explanation is that these people were exposed to TBEV naturally as the study period included spring and summer. We also hypothesized that exposure to SARS-CoV-2 and other pathogens had a potential boosting effect on TBEV titers. However, a post-hoc analysis of our data found no correlation between patient contact and up-regulation of TBEV antibodies, making this scenario less likely.

The dual methodological approach used in this study provides a unique insight into a large cohort of HCW exposed to COVID-19. Furthermore, the large sample size and prospective nature of this HCW cohort allowed us to correct for important co-variables. However, limitations include that previous TBEV vaccination was non-dated and self-reported and, as previously stated, that only the humoral but not the cellular immune response could be characterized. Also, the relatively small number of sera analysed for the presence of both SARS-CoV-2 and TBEV antibodies is a significant limitation of the study.

In conclusion, we found an association between previous TBEV vaccination and reduced seroconversion rates of anti-SARS-CoV-2 antibodies among a cohort of Swiss HCW. This observation could not be explained by characterizing the humoral adaptive immune response against either TBEV or SARS-CoV-2, as there was no cross-neutralizing effect of anti-TBEV antibodies against SARS-CoV-2 or vice versa. Although residual confounding is the most likely explanation, trained immunity, i.e., non-specific activation of the innate immune system conferring protection against heterologous infections, could also account for this finding although we did not observe a correlation to patient contact in this study. Since the beginning of the pandemic, many publications have hypothesized about potential cross-immunity against SARS-CoV-2 and despite the negative results reported here, we believe it is important to experimentally investigate such claims to separate facts from hypotheses.

## Methods

### Study design and sample sources

We analysed data from a prospective HCW cohort comprising 3352 participants from Northern and Eastern Switzerland^26^. In January and February 2021, participants were asked about previous vaccinations against TBEV (at least one) via online questionnaire. At the same time, venous blood samples were taken and analysed regarding the presence of anti-SARS-CoV-2 (anti-nucleocapsid) antibodies, using an electro-chemiluminescence immunoassay (ECLIA, Roche Diagnostics, Basel, Switzerland), as previously described^27^. Additionally, we analysed a convenience sample of baseline and follow-up sera from a precursor study of the above-mentioned cohort^28^, where participants had already provided baseline (March/April 2020, time point T1) and follow-up (August/September 2020, time point T2) blood samples. Blood samples were tested for the presence of anti-SARS-CoV-2 (anti-nucleocapsid) antibodies with the same ECLIA as previously described. For the laboratory analysis, three HCW groups were pre-defined: (i) HCW vaccinated against TBEV with seroconversion for SARS-CoV-2 between baseline and follow-up (group 1, n = 4); (ii) HCW vaccinated against TBEV without seroconversion for SARS-CoV-2 (group 2, n = 17); and (iii) HCW not vaccinated against TBEV without seroconversion and without positive PCR for SARS-CoV-2 (group 3, n = 5). Electronic informed consent was obtained from all study participants. The study was approved by the ethics committee of Eastern Switzerland (#2020–00,502), an institution of the health departments of the participating cantons (i.e. districts). All research was performed according to the relevant guidelines and regulations.

### Serum neutralization of SARS-CoV-2

To assess the presence of neutralizing antibodies against SARS-CoV-2, sera were heat-inactivated for 30 min at 56 °C and subsequently diluted 1:8 in Minimum Essential Medium (MEM; Seraglob, Schaffhausen, Switzerland) supplemented with 2% Foetal Bovine Serum (FBS; Seraglob, Schaffhausen, Switzerland). Further fivefold dilutions were made in duplicates in a 96-well plate (TPP Techno Plastic Products, Trasadingen, Switzerland) in a total volume of 50µL. One hundred TCID_50_ SARS-CoV-2 (2019-nCoV/IDF0372/2020, acquired from EVAg, Marseille, France) were added in equal volume. Sera and virus were incubated for 1 h at 37 °C before transfer to confluent Vero E6 cells (provided by Prof. Dr. Volker Thiel, University of Bern, Bern, Switzerland). Cells were then incubated at 37 °C, 5% CO_2_ and > 85% relative humidity (rH) for 72 h. After incubation, neutralization capacity was evaluated by crystal violet staining and reported as the GMT of two replicates.

### Serum neutralization of TBEV

Sera were heat-inactivated for 30 min. at 56 °C and subsequently diluted 1:8 in Leibovitz L-15 medium (Biochrom AG, Berlin, Germany) supplemented with 5% FBS (Seraglob, Schaffhausen, Switzerland). Further fivefold dilutions were made in duplicates in a 96-well plate (TPP Techno Plastic Products, Trasadingen, Switzerland) in a total volume of 50µL. One hundred TCID_50_ TBEV (Hypr, provided by Daniel Růžek, University of South Bohemia, České Budějovice, Czech Republic) were added in equal volume. Plates were incubated overnight at 4 °C and subsequently at 37 °C for 1 h without CO_2_. Porcine kidney stable (PS) cells (provided by Daniel Růžek, University of South Bohemia, České Budějovice, Czech Republic) were then added to each well (15,000 cells/well in 100µL), and plates further incubated at 37 °C without CO_2_. On day 3, neutral red dye in Dulbecco’s phosphate-buffered saline (Sigma Aldrich, Buchs SG, Switzerland) was added to each well at a final concentration of 0.000165%. On day 5, the liquid was removed, and the presence or absence of neutral-red-stained cells used to assess virus-induced cytopathic effect (CPE) and neutralizing capacity reported as the geometric mean titer (GMT) of two replicates.

In all neutralization tests, neutralization titer was defined as the reciprocal dilution resulting in at least 50% virus neutralization.

### TBEV ELISA

Sera were tested with SERION ELISA classic FSME Virus/TBE Virus IgG and IgM kits (VirionSerion GmbH, Würzburg, Germany). Analyses were performed manually according to the manufacturer’s instructions. Antibody titers (U/ml) were calculated with the following cut-offs: IgG negative < 100 U/ml, equivocal 100–150 U/ml, positive > 150 U/ml; IgM negative < 10 U/ml, equivocal 10–15 U/ml, positive > 15 U/ml).

### Statistical analysis

For the association between previous TBEV vaccination and positive SARS-CoV-2 serology, we used univariable and multivariable logistic regression. The following pre-defined variables were entered into the model: age, sex, profession (categories: nurse, physician, other), and patient contact (yes or no). SPSS statistical software, version 20.0 (IBM, Armonk, New York, USA) was used for the association analysis. For the comparison of antibody titers between baseline and follow-up sera, Wilcoxon matched-pairs signed rank test was applied using GraphPad Prism version 8.0.0 for Windows (GraphPad Software, San Diego, California USA www.graphpad.com). P-values < 0.05 were considered statistically significant.

## Data Availability

Data are available from the authors upon reasonable request.
